# Treatment outcomes for drug-resistant tuberculosis: a retrospective longitudinal study

**DOI:** 10.1186/s12879-025-11547-5

**Published:** 2025-09-25

**Authors:** Ruslan Akhmedullin, Gulbanu Algazyeva, Аnar Rakisheva, Gulnaz Mussabekova, Gulnur Zhakhina, Aigul Tursynbayeva, Abduzhappar Gaipov, Malik Adenov, Kulahmet Erimbetov, Shakhimurat Ismailov

**Affiliations:** 1 Department of Medicine, Nazarbayev University School of Medicine, Kerey and Zhanibek street 5/1, Astana, 010000 Kazakhstan; 2 National Center of Phthisiopulmonology Ministry of Health of the Republic of Kazakhstan, Almaty, Kazakhstan; 3 Kazakh National Medical University named after S. D.Asfendiyarov, Almaty, Kazakhstan; 4 Public Union “Kazakhstan Association of Phtisiopulmologists”, Almaty, Kazakhstan

**Keywords:** Multidrug-resistant tuberculosis, MDR-TB, Drug-resistant TB, Treatment effectiveness, Extensively drug-resistant tuberculosis, XDR-TB

## Abstract

**Background:**

This study examined the treatment outcomes of multidrug-resistant tuberculosis (MDR-TB) in Kazakhstan, where its burden is notably high.

**Methods:**

The authors conducted a retrospective longitudinal study using the National Tuberculosis Registry, this study analyzed treatment outcomes in MDR-TB patients from 2018 to 2021, and included adult patients (≥ 18 years) who completed a specific treatment. Outcomes were categorized into successful and unsuccessful treatments. Bivariate and multivariate Poisson regression models with modified errors were employed to obtain crude and adjusted risk ratios (aRR).

**Results:**

The study cohort comprised 12,698 cases, of which 10, 306 (81.16%) completed treatment with a successful outcome, while 2,392 (18.84%) had unsuccessful outcomes. Male sex (aRR 1.35, 95% CI 1.24–1.45), urban residency (aRR 1.16, 95% 1.07–1.24), having both extrapulmonary and pulmonary tuberculosis (aRR 1.49, 95% 1.04–2.15), XDR-TB (aRR 1.31, 95% 1.08–1.59), excessive alcohol consumption (aRR 1.43, 95% 1.28–1.59), HIV-positive status (aRR 2.24, 95% 2.01–2.47), and drug abuse (aRR 1.37, 95% 1.10–1.71) significantly elevated the risk of the unsuccessful treatment.

**Conclusion:**

Our findings underscore the need for focused strategies to reduce the MDR-TB burden, particularly among adults, male sex, relapsed cases, and XDR-TB. Despite the encouraging findings observed, further studies are necessary to update our estimates.

**Supplementary Information:**

The online version contains supplementary material available at 10.1186/s12879-025-11547-5.

## Background

Approximately a quarter of the world’s population is estimated to have Mycobacterium tuberculosis, up to 10% of which have lifetime risks of developing symptoms and TB [1]. Although tuberculosis (TB) is curable and preventable, it is a primary factor contributing to antimicrobial resistance-related deaths, making it a global health concern. In 2022, the World Health Organization (WHO) reported a decrease in TB cases from 7.1 million in 2019 to 5.8 million in 2020; however, in 2021, it began a new uptrend, reaching 6.4 million cases worldwide and raising concerns for population health [2]. Globally, low- and middle-income countries have a higher TB burden, with a specific emphasis on the Asian, Eastern European, and African regions [3, 4].

Kazakhstan is a Central Asian country. The nation has made progress towards universal health coverage [2]. Current findings suggest TB incidence to decline substantially during 2014–2019 [5]. Despite a reduction in the overall TB incidence, the country reports a high rate of multidrug-resistant TB (MDR-TB) [6] and remains on the WHO’s MDR-TB watch list [2].

Currently, MDR-TB is a major threat to global health, fueling the TB epidemic and worsening the health outcomes. The number of patients with MDR-TB has increased worldwide, necessitating improvements in TB control and treatment modalities [2]. Although Kazakhstan has ensured extensive universal health coverage, provided access to safe and effective medicines, and initiated various national TB programs [2, 5], the share of newly diagnosed MDR-TB remains markedly high [6, 7]. Specifically, rifampicin-resistant TB (MDR/RR-TB) accounts for 27% of incident TB cases and 44% of treated cases [8].

Although medications and treatment settings are largely available, patients with MDR-TB still face challenges in terms of adherence and tolerability, which complicates treatment progress. Hence, insufficient treatment contributes to a prolonged infection period, increasing both morbidity and mortality among patients with MDR-TB [9]. Understanding the factors affecting MDR-TB treatment outcomes is essential for evaluating therapeutic modalities, improving clinical practice, and developing public health strategies. However, several studies on TB treatment outcomes are available [10–12], and the evidence suggests heterogeneous reports. Hence, the growing concern over MDR-TB and the necessity to monitor refractory patients’ responses to treatment, especially in countries with a high MDR-TB burden, justify the need for literature updates. Thus, our study aimed to evaluate the treatment effectiveness and factors affecting these outcomes among patients with MDR-TB who received treatment in Kazakhstan between 2018 and 2021.

## Methods

### Overview and study design

This was a retrospective longitudinal study that utilized large-scale administrative data from the National Tuberculosis Registry (NTR) covering 2018–2021. Founded in 2001 by Kazakhstan’s Ministry of Health with support from the USAID, the NTR has adhered to international standards for TB reporting and data management since 2007. The NTR database integrates two additional national databases: the Electronic Register of Outpatients and the Register of Attached Population. Further information on these databases is available elsewhere [13].

### Study population and case definition

The study population consisted of DR-TB patients aged ≥ 18 years who had completed treatment with documented outcomes and detailed records in the NTR. All TB cases were confirmed using standard procedures based on local and WHO guidelines, including bacteriological assessments and drug resistance tests through molecular tests or laboratory cultivation techniques [14, 15]. In Kazakhstan, the Gene-Xpert test is the primary diagnostic method used for all patients with suspected TB. Along with this, other diagnostic means such as culture-based testing (Bactec, Löwenstein-Jensen) and molecular-genetic diagnostic tests (Hain-test, Bioneer) have been employed for diagnosis. The data on laboratory findings are available in the appendix (Supplementary Table 1).

MDR-TB was defined as resistance to both isoniazid (H) and rifampicin (R), based on either test. XDR-TB was defined as resistance to H, R, and a fluoroquinolone (levofloxacin [Lfx] or moxifloxacin [Mfx]) and at least one injectable drug (amikacin [Am], kanamycin [Km], or capreomycin [Cm]).

### Exposure and Covariates

The individual patient data included demographic and clinical information. The study entry date was January 1, 2018, and the endpoint of the follow-up period was December 31, 2021. We examined cases in which treatment began within the period between the study entry date and either the date of death or the end of the follow-up, whichever occurred first. Using the NTR, we were able to determine the treatment outcomes for all cases. Age at treatment initiation was determined as the difference in the treatment start date and date of birth. Subsequently, this variable was stratified into four categories: 18–44 years, 45–59 years, 60–74 years, and ≥ 75 years. Patients were classified into three groups based on their treatment history at entry: “New” for those newly diagnosed with TB, “Relapse” for those who began treatment after a relapse, and “Other”, which included patients who started treatment after a previous course had been stopped, failed, or interrupted.

### Outcome assessment

We used the National Standard TB definitions and 2021 WHO definition modifications for TB treatment outcomes (Supplementary Fig. 1) [9, 16]. Previous studies revealed that variable selection may result in unstable inferences that require substantially more cases to ensure adequate events per variable ratio and introduce systematic errors [17, 18]. Therefore, we did not pre-filter the variables or eliminate weak effects. Instead, we relied on expert background knowledge, including clinically important variable. The outcome of interest was unsuccessful treatment, which included treatment failure, loss to follow-up, and death. The predictors were treatment regimens, age, sex, residency, treatment mode, patient type at entry, TB localization, resistance (MDR/XDR), and clinical, behavioral, and social factors (e.g., excessive alcohol consumption, HIV status, diabetes mellitus, postpartum period, drug abuse, history of prison sentence, pregnancy). Response to treatment and toxicity were monitored through regular history taking, physical examination, chest radiography, and laboratory surveillance.

### Statistical analysis and methods

Baseline patient characteristics are presented as counts and percentages. We performed a exploratory data analysis of binary treatment outcomes. Given that, the prevalence of outcomes was common in our study cohort, bivariate and multivariate Poisson regression analyses with modified (robust) errors were employed to obtain crude and adjusted risk ratios. It is worth noting that the odds ratio often overestimates the effect size when the outcome is prevalent (e.g., > 10%), further requiring modified regression models to account for it [19]. P-values were two-sided and reported as statistically significant at *p* < 0.05. In addition, a sensitivity analysis was performed to exclude underpopulated groups. Coefficient estimates and variance inflation factors were used to detect multicollinearity, and a model fit was assessed with the Normalized Residual Sum of Squares [20], and information criterion and Bayesian information criterion were used to compare Logistic and Poisson regression models. We reported the final model that met these criteria. The only variable associated with missing values was “Destruction (on X-ray).” We generated another group called “Unknown” which accounted for 4.91% of the total cohort. The study was performed in accordance with the “STROBE” guidelines [21] (Supplementary Table 2). Data management, cleaning, and overall analyses were performed using STATA (version 18).

### Ethical consideration

This study used secondary data derived from the NTR with no direct involvement of the patients. Before the data were collected, the researchers provided signed confidentiality agreements and ensured that all data were confidential. This study was approved at a meeting of the Local Bioethics Commission of the National Scientific Center of Phthisiopulmonology of the Republic of Kazakhstan with the exception of informed consent (№ 3, August 2024).

## Results

We used data from 12,698 MDR-TB patients who received therapy nationwide between 2018 and 2021. Overall, MDR-TB was more common in males than in women in the study cohort (Table 1). The majority of patients (58.87%) belonged to the 18–44 age category. MDR-TB (97.28%), were newly diagnosed with TB (50.91%), had pulmonary TB (95.45%), resided in urban areas (62.39%), started intensive phase of inpatient treatment (78.96%), and received a special treatment regimen (54.24%) (Table 1 and Supplementary Table 1). The most prevalent clinical, behavioral, and social factors were excessive alcohol consumption (7.84%), followed by HIV (7.22%), diabetes (6.86%), and so forth (Supplementary Table 1).

**Table 1:** Baseline characteristics of study cohort

Covariate	Successful treatment (*n* = 10,*306; 81.16%*)	Unsuccessful treatment (n = *2*,*392; 18.84%*)	Total, (*n* = 12,*698; 100.00%*)
Age Categories, *n* (%)			
18–44 years old	6,413 (62.23%)	1,062 (44.40%)	7,475 (58.87%)
45–59 years old	2,656 (25.77%)	740 (30.94%)	3,396 (26.74%)
60–74 years old	1,043 (10.12%)	441 (18.44%)	1,484 (11.69%)
≥ 75 years old	194 (1.88%)	149 (6.23%)	343 (2.70%)
Sex, n (%)			
Female	3,595 (34.88%)	595 (24.87%)	4,190 (33.00%)
Male	6,711 (65.12%)	1,797 (75.13%)	8,508 (67.00%)
Residency, n (%)			
Urban	6,334 (61.46%)	1,588 (66.39%)	7,922 (62.39%)
Rural	3,972 (38.54%)	804 (33.61%)	4,776 (37.61%)
TB Localization, n (%)			
EPTB	369 (3.58%)	53 (2.22%)	422 (3.32%)
PTB	9,821 (95.29%)	2,299 (96.11%)	12,120 (95.45%)
EPTB + PTB	116 (1.13%)	40 (1.67%)	156 (1.23%)
Treatment mode, n (%)			
Inpatient	7,999 (77.61%)	2,027 (84.74%)	10,026 (78.96%)
Outpatient	2,307 (22.39%)	365 (15.26%)	2,672 (21.04%)
Treatment outcomes, n (%)			
Cured	5,721 (55.51%)	0 (0.00%)	5,721 (45.05%)
Treatment completed	4,585 (44.49%)	0 (0.00%)	4,585 (36.11%)
Died	0 (0.00%)	1,570 (65.64%)	1,570 (12.36%)
Lost to follow-up	0 (0.00%)	487 (20.36%)	487 (3.84%)
Treatment failure	0 (0.00%)	335 (14.01%)	335 (2.64%)

**Fig. 1:**
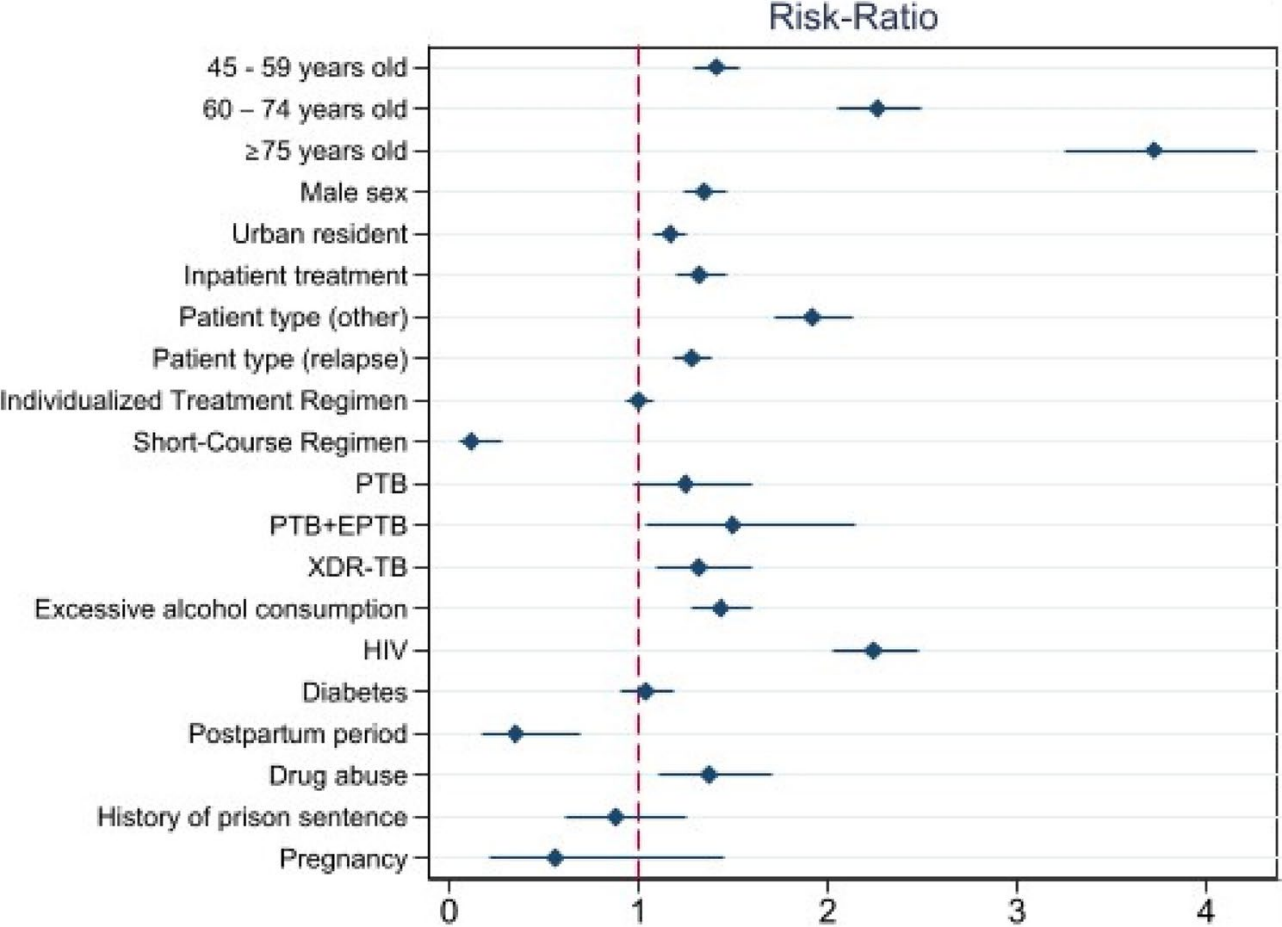
Association of covariates with treatment outcomes in MDR-TB patients. Note: Variance Inflation Factor = 1.93; Normalized RSS = 7.5%. The Information criterion and Bayesian information criterion favored Poisson regression.

As a result, out of 12,698 MDR TB patients, 10,306 (81.16%) patients achieved a successful treatment outcome, while 2,392 (18.84%) cases had unsuccessful treatment outcomes, of which 1,570 (65.64%) died, 487 (20.36%) were lost to follow-up, and 335 (14.01%) had treatment failure (Table 1). The crude and adjusted risk ratios (aRR) for the covariates are presented in Fig. 1 (Supplementary Table 3). In the adjusted estimates (Fig. 1, Supplementary Table 3), the individualized treatment regimen (aRR 1.00 [95% CI 0.93–1.07]) showed no significant difference compared to the special treatment regimen. However, patients on the short-course regimen had better outcomes, with more favorable estimates (aRR 0.11 [0.05–0.27]). In addition, increasing age, male sex (aRR 1.35 [1.24–1.45]), urban residency (aRR 1.16 [1.07–1.24]), inpatient treatment (aRR 1.32]1.19–1.46]), relapse patient type at entry (aRR 1.28 [1.18–1.38]), “other” patient type at entry (aRR 1.91 [1.72–2.12]), having both extrapulmonary and pulmonary tuberculosis (aRR 1.49 [1.04–2.15]), XDR-TB (aRR 1.31 [1.08–1.59]), excessive alcohol consumption (aRR 1.43 [1.28–1.59]), HIV-positive status (aRR 2.24 [2.01–2.47]), and drug abuse (aRR 1.37 [1.10–1.71]) were all significantly associated with an elevated risk for the unsuccessful treatment outcomes. In contrast, being in the postpartum period (aRR 0.35 [0.20–0.68]), having a history of imprisonment (aRR 0.87 [0.62–1.25]), and pregnancy (aRR 0.56 [0.22–1.45]) were negatively associated with unsuccessful outcomes; however, the latter two associations were not statistically significant. Positive associations between age and an unsuccessful outcome became stronger with each increasing age group: compared with participants aged 18–44 years, adjusted estimates were 1.41 (1.30–1.54) for those aged 45–59 years, 2.27 (2.06–2.51) for those aged 60–74 years, and 3.75 (3.27–4.30) for those aged 75 years and older.

## Discussion

This study evaluated the effectiveness of treatment in patients with DR-TB using the nationwide TB registry in Kazakhstan, where the burden of MDR-TB is notably high. The findings of our study revealed that the overall rate of unsuccessful treatment was 18.84%, which is lower than that reported in the WHO’s global estimates [22], ranging from 27.0 to 45.0%. We found that the rate significantly increased with age, as well as for relapse and “other” patient categories, but was less likely to occur in newly treated patients.

Kazakhstan has historically ranked among the countries with the highest DR-TB burden worldwide [22, 23]. To effectively grapple with its impact, the country has introduced the National Tuberculosis Program [11], established an efficient surveillance system to monitor epidemiological metrics through local tuberculosis dispensaries [24], and aggregated data in the National Tuberculosis Registry through the Unified National Electronic Healthcare System [13]. These efforts have led to a decline in the TB burden in the country; however, DR-TB continues to be a major public health concern in the country due to the emergence of MDR-TB strains. In 2018, the WHO recommended all-oral regimens for the treatment of MDR-TB [22]. Thus, the individualized treatment regimen did not reveal any differences in our study; however, our findings were in favor of the short-course regimen and agreed with the existing evidence [25–27], which contributed to its recommendation by WHO. In Kazakhstan, the Central Medical Advisory Committee decides who is eligible for the short-course regimen based on the local TB protocol [15]. The bacteriological results, radiologic findings, prior treatment history, and drug availability are considered when ascertaining a regimen. Although the short-course regimen group showed encouraging findings, they should be viewed as exploratory because they are based on a small number of events in a small subgroup. Further studies are required to confirm this association.

Successful treatment was defined as being cured or having completed treatment. Thus, better tolerance and lower adverse event rates than those of other regimens could partially explain this superiority [27]. In Kazakhstan, local guidelines recommend a short-course regimen for both MDR/XDR-TB patients who are resistant to fluoroquinolones and second-line injectable agents [15]. The scheme consists of an intensive period of 4–6 Bdq (6 months) – Lfx-Cfz-Z-E-Hh-Eto and a maintenance phase of 5 Lfx-Cfz-Z-E. With a shortened complete oral treatment regimen, the total course of treatment was 9–12 months. The prolonged treatment regimen included at least five TB drugs in groups A and B.

Notably, the short-course regimen had the smallest sample size in our study, of which only 0.21% completed treatment with an unsuccessful outcome. In other regimens, the average rate was 20.00%. Hence, in a post-hoc analysis, we excluded patients for whom a short-course regimen was assigned and compared the coefficients between the two models (Supplementary Fig. 2). This resulted in the estimates for all the covariates remaining unchanged. Nevertheless, future studies should closely examine the contribution of the short-course regimen when new data on treatment outcomes become available.

The WHO recommends favoring outpatient treatment, and Kazakhstan still has a high admission rate for inpatient TB centers [5, 11]. Interestingly, our findings suggest that the risk of unsuccessful treatment is higher in inpatient settings. The unsuccessful treatment outcomes in our study were primarily driven by death and loss to follow-up. When considered separately, both were more frequent in inpatient treatment. This is most likely justified by the fact that more complicated patients were hospitalized in inpatient facilities. Although the overall figures for these metrics were in line with those from WHO estimates [28, 29], future studies should thoroughly examine these associations.

The treatment success rate was significantly lower in patients with XDR-TB. Although these cases accounted for only 2.42% of our cohort, data from a recent study on DR-TB treatment outcomes in five post-Soviet Union countries suggest that our findings are natural [30]. Furthermore, for XDR-TB, our success rate was in line with the WHO’s goal of a 75.00% success rate [31]. Similar to MDR-TB, our findings were encouraging for both global estimates [32] and Kazakhstan, which was reported recently [33].

MDR-TB continues to pose a public health crisis and threat. In 2021, there were 450,000 incident MDR-TB cases globally, and the number of such cases is expected to increase [2]. This trend requires improvements in MDR-TB control, access, and treatment approaches. Continuous monitoring of MDR-TB treatment outcomes is, therefore, essential. Such vigilance helps policymakers evaluate the effectiveness of therapeutic interventions. Although our study revealed encouraging findings in terms of treatment success, further studies are still essential, specifically exploring the role of the short-course regimen, comparing the treatment modes, the impact of patient types on treatment outcomes, and collecting real-world data on safety parameters affecting treatment outcomes.

### Limitations

This study has several limitations. The major limitation was attributable to the use of secondary data that could underreport range of factors affecting treatment outcomes. For instance, underreported prevalence for some essential variables, such as HIV infection, duration of symptoms before treatment, and coexisting diseases, may affect our findings. Another limitation is that we were unable to draw clear inferences about the effectiveness of the treatment regimens, and the external validity may be weak because of the unequal distribution of participants across treatment regimens and the resistance type (MDR/XDR-TB), and exclusion of those aged less than 18 years. The lower rate of unsuccessful treatment for the short-course regimens is encouraging, but its generalizability is unclear.

Furthermore, we found that pregnancy and the postpartum state were associated with a lower risk of unfavorable treatment outcomes. This may be attributed to selection bias, with these groups representing only 0.57% and 1.85% of the total population, respectively. When sample size is small, inferences are extremely sensitive to small deviations, leading to ambiguous conclusions [34]. Hence, we may have model specification issues that require further investigation in these populations. Therefore, our findings for these categories require careful interpretation. Generalizability might be strengthened by more data on baseline resistance to other component drugs, and from prospective trials comparing short-course regimens to those of duration that is more conventional. Finally, because we used aggregated data and lacked detailed information on treatment regimens, we were unable to provide comprehensive information. Furthermore, we could not adjust for severity indicators (e.g., radiography, new vs. retreatment, and bedaquiline resistance) when comparing inpatient and outpatient treatment means and treatment outcomes in general. Therefore, our findings remain exploratory and should be interpreted with caution.

## Conclusion

In conclusion, our study presented MDR/XDR-TB treatment outcomes in Kazakhstan and discussed the determinants of outcomes. Overall, the unsuccessful treatment outcome rate in our study was lower than that reported by WHO. Unsuccessful TB treatment is more likely as age advances and among those with XDR-TB but is less likely in persons with newly diagnosed MDR-TB. The findings suggest targeted intervention is needed. Given the burden of MDR-TB in the country, future studies are needed to explore the effectiveness of treatment and validate our estimates.

## Supplementary Information

Supplementary Material 1.

## Data Availability

The datasets used and/or analyzed during the current study are available from the corresponding author on reasonable request.

## Abbreviations

DR-TB: Drug-resistant tuberculosis
MDR-TB: Multidrug-resistant tuberculosis
RR-TB: Rifampicin-resistant tuberculosis
XDR-TB: Extensively drug-resistant tuberculosis
USAID: United States Agency for International Development
NTR: National Tuberculosis Registry
STROBE: STrengthening the Reporting of OBservational studies in Epidemiology
HIV: Human immunodeficiency viruses
RR: Risk ratio
aRR: Adjusted risk ratios
H: Isoniazid
R: Rifampicin
Lfx: Levofloxacin
Mfx: Moxifloxacin
Am: Amikacin
Km: Kanamycin
Cm: Capreomycin
Bdq: Bedaquiline
Lnz: Linezolid
WHO: The World Health Organization
Z: Zidovudine
E: Ethambutol
Eto: Ethionamide
