# Supplementary material for: Treatment outcomes for drug-resistant tuberculosis: a retrospective longitudinal study
Source: BMC Infect Dis. 2025 Sep 25;25:1125. doi: 10.1186/s12879-025-11547-5 (PMC12466029; doi:10.1186/s12879-025-11547-5)
Supplement: Supplementary file 1 — Supplementary Material 1. [file 12879_2025_11547_MOESM1_ESM.docx]

**SUPPLEMENTS**

Supplementary Table 1. Baseline characteristics and laboratory findings of the study cohort;

Supplementary Table 2. STROBE Statement—Checklist;

Supplementary Table 3. Association of covariates with treatment outcomes (unsuccessful treatment) in MDR-TB patients.

Supplementary Figure 1. Outcome definitions for the study cohort;

Supplementary Figure 2. Model comparison.

Supplementary Table 1. Laboratory findings of the study cohort.

| **Covariate** | **Successful treatment**  **(*n=10,306; 81.16%*)** | **Unsuccessful treatment**  **(n=*2,392; 18.84%*)** | **Total, (*n=12,698; 100.0%*)** |
| --- | --- | --- | --- |
| ***Culture, n (%)*** | | | |
| ***Levenshtein-Jensen*** |  |  |  |
| Negative TB | 520 (5.05%) | 83 (3.47%) | 603 (4.75%) |
| Positive TB | 4,347 (42.18%) | 1,068 (44.65%) | 5,415 (42.64%) |
| Unknown | 5,439 (52.78%) | 1,241 (51.88%) | 6,680 (52.61%) |
| ***Bactec*** |  |  |  |
| Negative TB | 672 (6.53%) | 99 (4.14%) | 771 (6.08%) |
| Positive TB | 7,103 (68.99%) | 1,707 (71.45%) | 8,810 (69.46%) |
| ***Molecular-Genetic Diagnostic Tests, n (%)*** | | | |
| ***G-Xpert*** |  |  |  |
| TB Positive | 8,278 (80.32%) | 1,937 (80.98%) | 10,215 (80.45%) |
| TB Negative | 425 (4.12%) | 69 (2.88%) | 494 (3.89%) |
| Unknown | 1,603 (15.55%) | 386 (16.14%) | 1,989 (15.66%) |
| ***Bioneer*** |  |  |  |
| TB Positive | 731 (7.09%) | 152 (6.35%) | 883 (6.95%) |
| TB Negative |  |  |  |
| Unknown | 9,575 (92.91%) | 2,240 (93.65%) | 11,815 (93.05%) |
| ***Hain-test*** |  |  |  |
| TB Positive | 3,599 (34.92%) | 875 (36.58%) | 4,474 (35.23%) |
| TB Negative | 53 (0.51%) | 7 (0.29%) | 60 (0.47%) |
| Unknown | 6,654 (64.56%) | 1,510 (63.13%) | 8,164 (64.29%) |
| ***TB confirmation, n (%)*** | | | |
| Bacteriologically confirmed | 10,232 (99.28%) | 2,381 (99.54%) | 12,613 (99.33%) |
| Clinically confirmed | 74 (0.72%) | 11 (0.46%) | 85 (0.67%) |
| ***Patient’s type at entry, n (%)*** | | | |
| New | 5,531 (53.67%) | 934 (39.05%) | 6,465 (50.91%) |
| Relapse | 3,910 (37.94%) | 1,096 (45.82%) | 5,006 (39.42%) |
| Other | 865 (8.39%) | 362 (15.13%) | 1,227 (9.66%) |
| ***Treatment regimen, n (%)*** | | | |
| Special Treatment Regimen | 5,568 (54.03%) | 1,320 (55.18%) | 6,888 (54.24%) |
| Individualized Treatment Regimen | 4,416 (42.85%) | 1,067 (44.61%) | 5,483 (43.18%) |
| Short-Course Regimen | 322 (3.12%) | 5 (0.21%) | 327 (2.58%) |
| ***Destruction (on X-ray), n (%)*** | | | |
| Yes | 5,233 (50.78%) | 1,397 (58.40%) | 6,630 (52.21%) |
| No | 4,556 (44.21%) | 888 (37.12%) | 5,444 (42.87%) |
| Unknown | 517 (5.02%) | 107 (4.47%) | 624 (4.91%) |
| ***Resistance, n (%)*** | | | |
| MDR | 10,081 (97.82%) | 2,310 (96.57%) | 12,391 (97.58%) |
| XDR | 225 (2.18%) | 82 (3.43%) | 307 (2.42%) |
| ***Clinical, behavioral, and social factors, n (%)*** | | | |
| Excessive alcohol consumption | 704 (6.83%) | 291 (12.17%) | 995 (7.84%) |
| HIV | 574 (5.57%) | 343 (14.34%) | 917 (7.22%) |
| Diabetes | 687 (6.67%) | 184 (7.69%) | 871 (6.86%) |
| Postpartum period | 227 (2.20%) | 8 (0.33%) | 235 (1.85%) |
| Drug abuse | 89 (0.86%) | 54 (2.26%) | 143 (1.13%) |
| History of prison sentence | 99 (0.96%) | 24 (1.00%) | 123 (0.97%) |
| Pregnancy | 68 (0.66%) | 4 (0.17%) | 72 (0.57%) |

Supplementary Table 2. STROBE Statement—Checklist.

|  | Item No | Recommendation |
| --- | --- | --- |
| **Title and abstract** | 1 | (*a*) Indicate the study’s design with a commonly used term in the title or the abstract |
|  |  | (*b*) Provide in the abstract an informative and balanced summary of what was done and what was found |
| Introduction | | |
| Background/rationale | 2 | Explain the scientific background and rationale for the investigation being reported |
| Objectives | 3 | State specific objectives, including any prespecified hypotheses |
| Methods | | |
| Study design | 4 | Present key elements of study design early in the paper |
| Setting | 5 | Describe the setting, locations, and relevant dates, including periods of recruitment, exposure, follow-up, and data collection |
| Participants | 6 | (*a*) Give the eligibility criteria, and the sources and methods of case ascertainment and control selection. Give the rationale for the choice of cases and controls |
|  |  | (*b*) For matched studies, give matching criteria and the number of controls per case |
| Variables | 7 | Clearly define all outcomes, exposures, predictors, potential confounders, and effect modifiers. Give diagnostic criteria, if applicable |
| Data sources/ measurement | 8* | For each variable of interest, give sources of data and details of methods of assessment (measurement). Describe comparability of assessment methods if there is more than one group |
| Bias | 9 | Describe any efforts to address potential sources of bias |
| Study size | 10 | Explain how the study size was arrived at |
| Quantitative variables | 11 | Explain how quantitative variables were handled in the analyses. If applicable, describe which groupings were chosen and why |
| Statistical methods | 12 | (*a*) Describe all statistical methods, including those used to control for confounding |
|  |  | (*b*) Describe any methods used to examine subgroups and interactions |
|  |  | (*c*) Explain how missing data were addressed |
|  |  | (*d*) If applicable, explain how matching of cases and controls was addressed |
|  |  | (*e*) Describe any sensitivity analyses |
| Results | | |
| Participants | 13* | (a) Report numbers of individuals at each stage of study—eg numbers potentially eligible, examined for eligibility, confirmed eligible, included in the study, completing follow-up, and analysed |
|  |  | (b) Give reasons for non-participation at each stage |
|  |  | (c) Consider use of a flow diagram |
| Descriptive data | 14* | (a) Give characteristics of study participants (eg demographic, clinical, social) and information on exposures and potential confounders |
|  |  | (b) Indicate number of participants with missing data for each variable of interest |
| Outcome data | 15* | Report numbers in each exposure category, or summary measures of exposure |
| Main results | 16 | (*a*) Give unadjusted estimates and, if applicable, confounder-adjusted estimates and their precision (eg, 95% confidence interval). Make clear which confounders were adjusted for and why they were included |
|  |  | (*b*) Report category boundaries when continuous variables were categorized |
|  |  | (*c*) If relevant, consider translating estimates of relative risk into absolute risk for a meaningful time period |

| Other analyses | 17 | Report other analyses done—eg analyses of subgroups and interactions, and sensitivity analyses |
| --- | --- | --- |
| Discussion | | |
| Key results | 18 | Summarise key results with reference to study objectives |
| Limitations | 19 | Discuss limitations of the study, taking into account sources of potential bias or imprecision. Discuss both direction and magnitude of any potential bias |
| Interpretation | 20 | Give a cautious overall interpretation of results considering objectives, limitations, multiplicity of analyses, results from similar studies, and other relevant evidence |
| Generalisability | 21 | Discuss the generalisability (external validity) of the study results |
| Other information | | |
| Funding | 22 | Give the source of funding and the role of the funders for the present study and, if applicable, for the original study on which the present article is based |

Supplementary Table 3. Association of covariates with treatment outcomes (unsuccessful treatment) in MDR-TB patients.

| **Covariate** | **Crude Risk-Ratio** | **p-value** | **Adjusted Risk-Ratio** | **p-value** |
| --- | --- | --- | --- | --- |
| *Age category* | | | | |
| 18 - 44 years old | 1.0 |  | 1.0 |  |
| 45 - 59 years old | 1.53 (1.41-1.66) | <0.001 | 1.41 (1.30-1.54) | 0.001 |
| 60 – 74 years old | 2.10 (1.91-2.30) | <0.001 | 2.27 (2.06-2.51) | <0.001 |
| ≥75 years old | 3.05 (2.67-3.49) | <0.001 | 3.75 (3.27-4.30) | <0.001 |
| *Gender* | | | | |
| Female | 1.0 |  | 1.0 |  |
| Male | 1.49 (1.36-1.62) | <0.001 | 1.35 (1.24-1.45) | <0.001 |
| *Residency* | | | | |
| Rural | 1.0 |  | 1.0 |  |
| Urban | 1.20 (1.12-1.28) | <0.001 | 1.16 (1.07-1.24) | <0.001 |
| *Treatment mode* | | | | |
| Outpatient | 1.0 |  | 1.0 |  |
| Inpatient | 1.19 (1.10-1.28) | <0.001 | 1.32 (1.19-1.46) | <0.001 |
| *Patient’s type at entry* | | | | |
| New | 1.0 |  | 1.0 |  |
| Relapse | 1.51 (1.40-1.64) | <0.001 | 1.28 (1.18-1.38) | <0.001 |
| Other | 2.04 (1.84-2.26) | <0.001 | 1.91 (1.72-2.12) | <0.001 |
| *Treatment regimen* | | | | |
| Special Treatment Regimen | 1.0 |  | 1.0 |  |
| Individualized treatment regimen | 1.01 (0.94-1.09) | 0.67 | 1.00 (0.93-1.07) | 0.98 |
| Short course regimens | 0.10 (0.03-0.19) | <0.001 | 0.11 (0.05-0.27) | <0.001 |
| *Localization* | | | | |
| EPTB | 1.0 |  | 1.0 |  |
| PTB | 1.51 (1.17-1.94) | 0.001 | 1.24 (0.97-1.59) | 0.08 |
| EPTB+PTB | 2.04 (1.41-2.95) | <0.001 | 1.49 (1.04-2.15) | 0.03 |
| *Resistance* | | | | |
| MDR | 1.0 |  | 1.0 |  |
| XDR | 1.43 (1.18-1.73) | <0.001 | 1.31 (1.08-1.59) | 0.01 |
| *Risk factors* | | | | |
| Excessive alcohol consumption | 1.63 (1.46-1.81) | <0.001 | 1.43 (1.28-1.59) | <0.001 |
| HIV | 2.15 (1.96-2.35) | <0.001 | 2.24 (2.01-2.47) | <0.001 |
| Diabetes | 1.13 (0.98-1.29) | 0.07 | 1.03 (0.90-1.19) | 0.63 |
| Postpartum period | 0.17 (0.10-0.35) | <0.001 | 0.35 (0.20-0.68) | 0.002 |
| Drug abuse | 2.02 (1.63-2.51) | <0.001 | 1.37 (1.10-1.71) | <0.001 |
| History of prison sentence | 1.03 (0.72-1.48) | 0.85 | 0.87 (0.62-1.25) | 0.48 |
| Pregnancy | 0.29 (0.11-0.76) | 0.01 | 0.56 (0.22-1.45) | 0.23 |


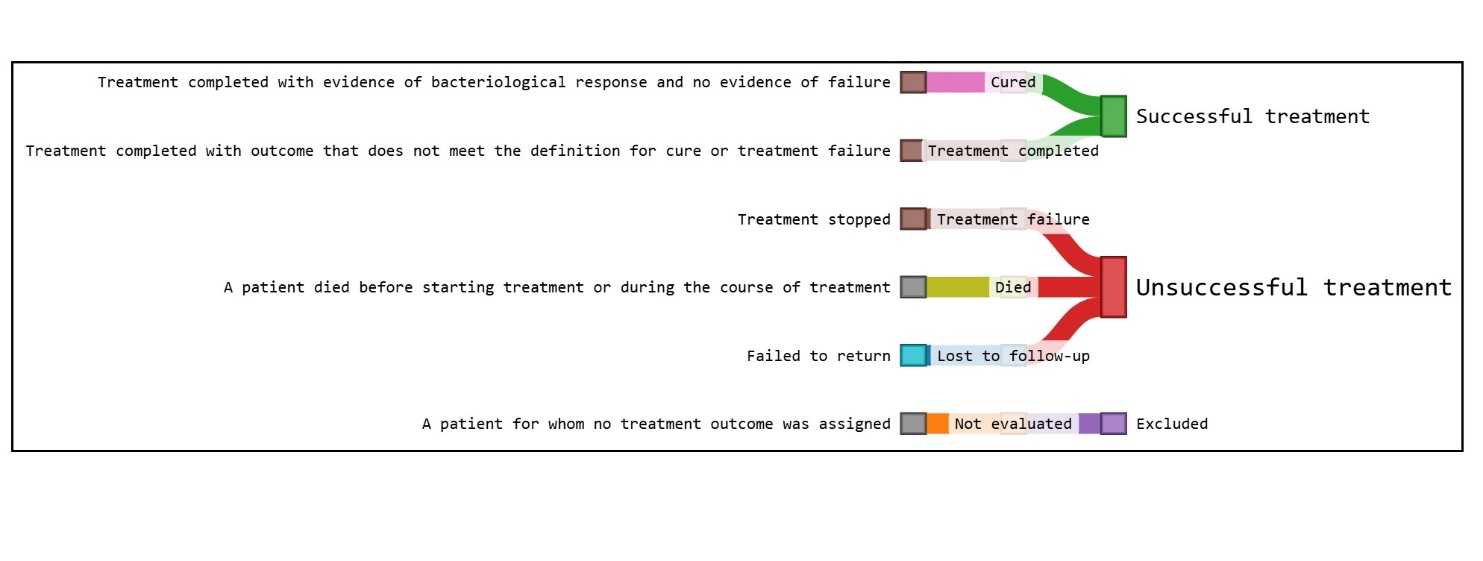
Supplementary Figure 1. Outcome definitions for the study cohort.

Supplementary Figure 2. Model comparison.


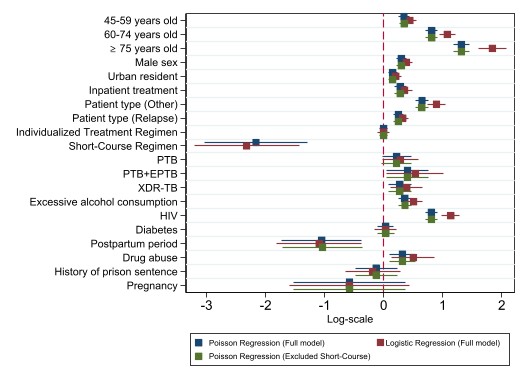


**Information criterion and Bayesian information criterion favored Poisson regression.*
